# Fatty Acid Composition and Aromatic Profile of Krškopolje and Modern Pig Breeds Reared Under Organic and Conventional Systems

**DOI:** 10.3390/foods15050866

**Published:** 2026-03-04

**Authors:** Marjeta Mencin, Katja Babič, Lidija Strojnik, Zala Sel, Andrej Kastelic, Nives Ogrinc

**Affiliations:** 1 Department of Environmental Sciences, Jožef Stefan Institute, Jamova 39, 1000 Ljubljana, Slovenia; marjeta.mencin@ijs.si (M.M.); katja.babic93@gmail.com (K.B.); lidija.strojnik@ijs.si (L.S.); zala.sel@ijs.si (Z.S.); 2 Jožef Stefan International Postgraduate School, Jamova 39, 1000 Ljubljana, Slovenia; 3 Chamber of Agriculture and Forestry of Slovenia, KGZ Novo Mesto, Šmihelska 14, 5000 Novo Mesto, Slovenia; andrej.kastelic@kgzs-zavodnm.si; 4 Research and Innovation Center Prekmurje, Jožef Stefan Institute, Lendavska Ulica 5a, 9000 Murska Sobota, Slovenia

**Keywords:** Krškopolje pigs, volatile organic compounds, fatty acids, organic production, conventional production, multivariate analysis

## Abstract

Slovenia preserves one autochthonous pig breed, the Krškopolje pig, whose meat has been reported to exhibit a favourable fatty acid profile compared with that of modern breeds. However, meat quality is not solely determined by genetics; the production system also influences it, as organic and conventional farming differ in feed composition, housing and outdoor access. This study aimed to compare the effects of pig breed (Krškopolje vs. modern) and production system (organic vs. conventional) on the fatty acid composition and volatile organic compound (VOC) profile of pork. Fatty acid composition was determined by GC-FID after methylation, and the VOCs profile was obtained using headspace solid-phase microextraction (HS-SPME) coupled with GC-MS. Results showed that Krškopolje meat had higher SFA and MUFA, while modern pig meat had higher PUFAs, particularly n-6, reflecting genetic and dietary influences. Modern breeds also showed greater fatty acid response to the rearing system than the Krškopolje breed. Several VOCs were unique to modern breed pigs, indicating breed-specific differences in lipid composition, amino acid metabolism, and microbial activity. Aldehydes were the dominant VOC class in both breeds, slightly higher in Krškopolje meat. OPLS-DA model revealed breed-related differences in VOCs, pinpointing compounds likely responsible for breed-specific aroma and flavour.

## 1. Introduction

In Slovenia, the only recognized autochthonous pig breed is the Krškopolje, which originated in the Dolenjska region. Renowned for its high-quality meat, with a more favourable fatty acid profile than modern pig breeds, this breed is well adapted to poor feeding and challenging environmental conditions, exhibits average fertility, a good appetite, and high growth rate, and represents an important component of biodiversity and the conservation of autochthonous breeds [1]. In contrast, modern pig breeds have been genetically selected for rapid growth and lean meat deposition, resulting in differences from autochthonous breeds in fat deposition, fat metabolic characteristics, and other meat properties [2].

Flavour is a critical quality parameter for pork, and increasing consumer interest in flavour makes it an important determinant of pork selection [3]. Pig breed is considered one of the key factors directly affecting intramuscular fat (IMF) content, which positively influences flavour quality [4,5]. Lipid oxidation and hydrolysis are major biochemical processes involved in the formation of pork aroma. Endogenous factors, including enzymes, metabolism, and fatty acid composition, as well as exogenous factors such as processing, storage, and microbial activity, gradually break down lipids into free fatty acids. These free fatty acids influence meat firmness, oxidative stability, shelf life and nutritional properties [6]. They also serve as important flavour precursors, as they are further converted into volatile organic compounds (VOCs), including ketones, aldehydes, alcohols, esters, furans, and lactones, which contribute significantly to meat flavour [4].

Meat quality is not only determined by intrinsic factors such as breed, but also by extrinsic factors associated with production systems. Organic and conventional meat production systems differ in multiple aspects, including feed composition, space allowance, floor type, access to straw when indoors, and outdoor access [5]. Krškopolje pigs are predominantly raised on small-scale farms, often organic or agrotourism farms [7]. In contrast, most modern breed pigs are produced in intensive, conventional systems that use slatted-floor housing and modern feeding techniques to produce lean pork at a reasonable price [8].

Organic pig farming, regulated by EU legislation (Regulation (EU) 2018/848), represents the most controlled alternative housing system, ensuring high animal welfare standards, environmentally sustainable production, chemical residue-free pork, with specific feed requirements, including at least 30% of feed sourced from the farm itself or local organic production units [9]. These production differences can influence meat quality, particularly IMF content, which tends to be higher in organic pork compared to conventional meat [10,11,12].

Diets low in amino acids can enhance IMF without promoting excessive fat deposition, a consideration particularly relevant in organic production systems where synthetic amino acids and genetically modified feed ingredients are restricted, making diet a key determinant of IMF [13]. Moreover, feed formulation affects growth performance and several meat and fat quality characteristics, including its polyunsaturated fatty acid (PUFA) content [13]. Moreover, consumers often perceive organic products as healthier, safer and more nutritious than meat from intensive conventional pig production [8].

According to data from the Statistical Office of the Republic of Slovenia, the average Slovenian consumed approximately 89 kg of meat in 2021, with pork representing the largest share (about 33 kg per capita) [14]. Despite the importance of pork in the national diet, only a few studies have investigated the quality of pork derived from modern and autochthonous pig breeds reared under organic and conventional production systems.

This study evaluated the combined effects of pig breed (Krškopolje vs. modern) and production system (organic vs. conventional) on fatty acid composition and VOC profiles in pork. It also sought to determine how genetic background and production systems influence key meat quality traits. In doing so, the study provides a scientific basis for strategies to improve pork quality and promote the sustainable use of autochthonous pig breeds.

## 2. Materials and Methods

### 2.1. Samples

Raw pork samples were collected from the *Longissimus dorsi* muscle sourced from various Slovenian suppliers in 2022, 2024, and 2025. All animals were slaughtered within comparable production cycles and during the same season (winter). Feeding regimens within each production system (organic vs. conventional) followed standard farm practices that were generally consistent across years. However, minor variations in feed composition may have occurred due to agricultural and meteorological variability.

Samples were collected from two different pig breeds: the autochthonous Krškopolje breed and modern breed pigs. These were categorized by rearing system as either conventional (CON) or organic (ORG). The sample sizes for fatty acid composition analysis were as follows: 71 samples from Krškopolje pigs (35 CON, 36 ORG) and 74 samples from modern breed pigs (70 CON, 4 ORG). The modern breed ORG group included only four samples due to limited availability of organically reared modern pigs in Slovenia and constraints on farm participation. Pork samples from Krškopolje pigs were transported to the lab on the same day or the day after slaughter from a Slovenian farm. Modern breed samples were either collected within a day of slaughter from local farms or sourced from the Slovenian market.

### 2.2. Fatty Acid Analysis by GC-FID

Homogenized pork meat (0.5 g) was placed into a 12 mL glass vial and mixed with 500 µL of dichloromethane (CH_2_Cl_2_, J.T. Baker B.V., Deventer, The Netherlands). The mixture was extracted in an ultrasonic bath for 15 min. After extraction, 3 mL of 0.5 M NaOH (ACS reagent, ≥98%, pellets; Sigma-Aldrich, St. Louis, MO, USA) in methanol (J.T. Baker B.V., Deventer, Netherlands) was added, and the headspace was flushed with nitrogen gas. The sealed vials were heated at 90 °C for 10 min to promote saponification. After cooling in an ice-water bath, fatty acids were methylated by adding 3 mL of 14% BF_3_–MeOH solution (Sigma-Aldrich, St. Louis, MO, USA) and heating at 90 °C for 10 min. After cooling, the fatty acid methyl esters (FAMEs) were extracted with 1.5 mL of hexane (J.T. Baker B.V., Deventer, The Netherlands). The organic phase was transferred into GC vials and stored at −80 °C until analysis.

The characterization of FAMEs was performed using an Agilent 6890N Gas Chromatograph fitted with a Flame Ionization Detector (GC-FID; Agilent Technology, Santa Clara, CA, USA). GC-FID is an analytical technique often used to separate and quantify individual fatty acids after conversion to FAMEs. Separation was achieved on an Omegawax 320 capillary column (30 m × 0.32 mm, 0.25 µm film thickness) (Supelco, Bellefonte, PA, USA). The temperature programme was as follows: 50 °C (2 min hold) to 220 °C at 4 °C/min (20 min hold). Helium was used as the carrier gas in constant-flow mode (1 mL/min). Injector and detector temperatures were set at 260 °C and 280 °C, respectively.

The individual fatty acids were identified and quantified by comparing their retention times with those of a standard Supelco 37-component FAME Mix (Supelco) and expressed as weight per cent of total fatty acids. Procedural blanks were included in each analytical set of samples, and the Standard Supelco 37-component FAME mixture was analyzed after every 10 samples to verify instrument stability. Method precision based on measurements of replicate (*n* = 2) real samples was 5%. Results were expressed as the percentage of each fatty acid with respect to the total fatty acids [10]. This percentage was calculated from the corrected peak areas of the samples using the respective correction factors, as described by Christie and Han [11].

#### Calculation of Atherogenicity (AI) and Thrombogenicity (TI) Indices

The AI represents the ratio between the sum of the main pro-atherogenic fatty acids, including saturated fatty acids (SFAs) with 12, 14, or 16 carbon atoms and trans-fatty acids (TFAs), and the main classes of anti-atherogenic unsaturated fatty acids, including monounsaturated fatty acids (MUFAs) and polyunsaturated fatty acids (PUFAs). The TI is associated with a tendency to form blood clots in blood vessels and is defined as the ratio of pro-thrombogenic fatty acids (stearic acid [C18:0], myristic acid [C14:0], palmitic acid [C16:0]), TFAs to anti-thrombogenic fatty acids (MUFAs, PUFAs).

The AI and TI were calculated according to Equations (1) and (2), respectively, as proposed by Ulbricht and Southgate [12].

(1) $$AI=\frac{\left(C_{\left\{12:0\right\}}+4*C_{\left\{14:0\right\}}+C_{\left\{16:0\right\}}+TFA\right)}{\left(MUFA+n\text{-}6+n\text{-}3\right)}$$

(2) $$TI=\frac{\left(C_{\left\{14:0\right\}}+C_{\left\{16:0\right\}}+C_{\left\{18:0\right\}}+TFA\right)}{\left(0.5*MUFA+0.5*\left(n\text{-}6\right)+3*\left(n\text{-}3\right)+\left(\frac{n\text{-}3}{n\text{-}6}\right)\right)}$$

TFAs were added to the totals of the individual SFAs because of their similar atherogenic properties to SFAs. Since myristic acid is four times more atherogenic than other SFAs, it is assigned a coefficient of 4. As the PUFA n-6 fatty acids and MUFAs are less antithrombogenic than the n-3 fatty acids, they were assigned a coefficient of 0.5. The n-3 fatty acids were assigned a coefficient of 3 [15].

### 2.3. Volatile Compound Analysis by HS-SPME/GC-MS

VOC profiling was performed on a representative subset of samples collected in 2024: 10 samples from the Krškopolje breed (organically produced) and 21 samples from the modern breed (conventional production). Selection was based on the fatty acid dataset, and the chosen samples reflected the range of fatty acid compositions observed within each breed. Since rearing of the Krškopolje breed in Slovenia is limited to approximately 100 farms, sampling was structured to include approximately every 10th farm to maximize the representativeness of available production units.

Headspace solid-phase microextraction (HS-SPME) coupled with GC-MS was used for VOC profiling, following an in-house-validated method described by Babič et al. [16]. HS-SPME/GC-MS is an analytical technique used for the extraction, separation, and identification of VOCs. It enables sensitive profiling of aroma-related compounds by concentrating volatiles from the sample headspace prior to GC separation and MS detection [16]. Before each extraction, the SPME fibre (50/30 μm DVB/CAR/PDMS, 2 cm, Supelco, Bellefonte, PA, USA) was conditioned for 5 min at 250 °C, and reconditioned for 20 min after each analysis. For extraction, 1 g of homogenized meat sample was weighed into a 10 mL glass vial (Supelco), followed by the addition of 1 mL saturated NaCl solution and 50 μL of toluene-d8 (1.2 mg/mL) as internal standard. Vials were capped with a silicone/PTFE septum. The samples were extracted using HS-SPME conditions, with a 60 min equilibration time, a 60 min extraction time and a 70 °C equilibration and extraction temperature. Desorption of analytes was performed in the GC injector at 250 °C for 4 min using a straight Ultra Inert SPME Liner (Sigma-Aldrich, Supelco, USA).

The GC-MS analyses were performed using an Agilent 7890B GC coupled to a 5977A mass selective detector (MSD) (Agilent Technologies, USA). Separation was achieved on a VF-WAXms capillary column (30 m × 0.25 mm i.d. × 0.25 μm film thickness, Agilent Technologies, Santa Clara, CA, USA). Helium was used as carrier gas at a constant flow of 1.5 mL/min, and injections were performed in splitless mode. The temperature programme was as follows: initially, 40 °C for 1 min, then increased to 150 °C at 6 °C/min, to 200 °C at 10 °C/min, and finally to 250 °C at 20 °C/min, with a 10 min hold. Total run time was 37 min. The transfer line, ion source, and quadrupole temperatures were 280 °C, 240 °C, and 180 °C, respectively. Electron ionization (EI) was performed at 70 eV with an *m*/*z* scan range of 35–300 at a scan rate of 5.2 scans s^−1^ (full scan mode).

Data were acquired using ChemStation software (version F.01.03.2357, Agilent Technologies, Santa Clara, CA, USA). Identification was performed using spectral similarity with the NIST Spectral Database 14 (Agilent, USA) and matching retention indices (RIs) based on n-alkanes (C9–C23). The confirmation of tentatively identified compounds was made by comparing the calculated RIs according to the equation of Van Den Dool and Kratz [17], with those available in the NIST Chemistry WebBook, SRD 69, and PubChem (normal alkane RI, polar column and custom temperature program). Each identified compound was expressed as a relative percentage, calculated by normalizing its peak area to the total area of all identified peaks using toluene-d8 as the internal standard. This approach provides a semi-quantitative profile of volatile composition.

### 2.4. Statistical Analysis

Statistical analysis included a nonparametric Kruskal–Wallis test, performed with XLSTAT 2024 (Addinsoft, Long Island, NY, USA). Probability (*p*) values < 0.05 were used to indicate statistical significance. The plots were generated using RStudio (version 2023.12.1). Orthogonal Partial Least Squares Discriminant Analysis (OPLS-DA) was used to classify samples based on the VOC profiles. The analysis was performed using SIMCA (version 15, Sartorius Stedim Biotech, Umeå, Sweden).

## 3. Results and Discussion

### 3.1. Fatty Acid Composition of Krškopolje vs. Modern Breed Samples

The fatty acid composition of meat differed significantly between Krškopolje and modern pig breeds (Table 1), indicating a strong breed effect on lipid metabolism and fat deposition. Overall, Krškopolje breed meat was characterized by significantly higher proportions of SFA and MUFA, by 3.7% and 7.3%, respectively, whereas modern breed meat contained significantly higher levels of PUFA (39.8%), particularly n-6 PUFA.

Significant breed-related differences were also observed for individual SFAs, including short- and long-chain fatty acids (C12:0, C20:0–C24:0), with Krškopolje meat generally exhibiting higher values. Palmitic acid (C16:0), the predominant SFA in pork, was also significantly higher in Krškopolje meat (*p* = 0.017), while stearic acid (C18:0) showed a similar value (*p* = 0.069) to that of modern breed meat. Higher SFA levels in Krškopolje pigs may be related to their genotype, slower growth rate, and higher IMF content, which favours de novo synthesis and accumulation of SFA and MUFA [18].

Similar patterns have been reported for other local pig breeds compared with modern breeds [2,19]. Wood et al. [18] further suggested that the higher SFA content observed in Krškopolje pigs may be explained by higher carcass adiposity. Consistent with a higher muscle fat deposition, local pig breeds typically exhibit higher lipogenic enzyme activity and lower lipolytic enzyme activity than modern breeds. Several studies comparing the lipogenic potential of autochthonous and modern pig breeds have demonstrated enhanced lipogenic and desaturation capacity, along with reduced lipolysis, in autochthonous breeds [20,21].

Oleic acid (C18:1 cis-9), the dominant MUFA, was significantly higher in Krškopolje meat (7.0%) than in modern breed meat. Several minor MUFAs (C14:1, C17:1, C20:1, and C24:1) were also present at significantly higher levels in the Krškopolje breed. Higher MUFA levels, particularly oleic acid, are commonly associated with autochthonous pig breeds and are considered beneficial for meat quality, sensory attributes, and oxidative stability [22]. According to the literature, the characteristic fatty acid profile is mainly attributed to greater de novo synthesis of SFA and higher desaturation capacity leading to increased MUFAs [18,23].

In contrast, meat from modern breeds had significantly higher total PUFA levels than Krškopolje meat. This difference was due to the higher n-6 PUFA content in modern breeds, particularly linoleic acid (C18:2 n-6), which was for 48.2% higher in modern breed meat than in Krškopolje meat. The higher PUFA content, especially n-6 fatty acids, observed in modern pig breeds is largely attributed to genetic selection for lean growth and greater incorporation of plant-based feeds rich in linoleic acid, as PUFAs cannot be synthesized de novo by pigs and are obtained exclusively from the diet [2,18]. However, modern genotypes with higher PUFA proportions are also more susceptible to lipid oxidation.

Even though the differences in fatty acids are statistically significant but numerically small, differences in content of individual SFAs and MUFAs between Krškopolje and modern pigs are primarily breed-related and influence practical traits such as IMF content, tenderness, juiciness, and flavour, which are valued in high-quality pork. Variations in individual PUFAs content, especially n-6 and n-3 fatty acids, are largely diet-dependent, affecting the n-6:n-3 ratio and nutritional quality of meat [2,18].

The n-6:n-3 ratio was more favourable in Krškopolje meat than in modern pig meat, suggesting a nutritionally better fatty acid balance in the autochthonous breed (Table 1). The results agree with those of Škrlep et al. [24], who also reported that Krškopolje meat had a lower n-6:n-3 ratio than modern breed meat. Although a lower ratio may be beneficial for health, particularly in reducing cardiovascular risk, it should be emphasized that the values observed in the present study remain very high (>15). In contrast, the recommended ratio is <4 [24]. In general, studies confirm that pork is characterized by a high n-6:n-3 PUFA ratio due to conventional feeding practices [25].

In Slovenia, pig diets are based primarily on home-grown cereals, predominantly wheat, barley, and corn, along with young grass, clover, cooked root crops and soybean, resulting in diets rich in n-6 PUFA and relatively low in n-3 PUFA [26]. Dietary supplementation with linseed or microalgae has been shown to increase n-3 PUFA content in pork [27], offering a practical strategy to improve the n-6:n-3 ratio while preserving breed-specific quality traits. Such an approach may be particularly relevant for Krškopolje pigs, enhancing their nutritional profile without compromising their characteristic sensory properties and IMF content.

Fatty acids can also exert positive or negative effects on atherosclerosis and coronary thrombosis, depending on their degree of saturation and the geometric positions of the double bonds. The AI and TI take into account the different effects of individual fatty acids on human health, particularly on the increasing incidence of atheroma or thrombus formation [12]. An AI value below 0.5 is considered optimal, with no strict limit set for TI; values close to or below 1.0 are generally considered favourable. In the present study, the slightly higher AI and TI observed in Krškopolje meat reflect its higher SFA content. In contrast, the lower indices in modern pig meat are associated with higher PUFA levels. Nevertheless, the AI values in both pig breeds were within the recommended limit, while TI was slightly above the suggested limit.

Overall, Krškopolje pork is characterized by higher SFA and MUFA and lower PUFA contents, reflecting its traditional genotype and lipid metabolism. In contrast, modern pork contains higher PUFAs, particularly *n*-6 fatty acids, due to genetic selection and diet. Krškopolje meat also exhibits higher AI and TI values than those of modern breeds, yet it is recognized as high-quality pork. This contrast arises because high quality is primarily determined by sensory attributes, such as tenderness, juiciness, flavour, and aroma, which are likely associated with higher IMF content [2,9].

#### 3.1.1. Discrimination Between Krškopolje and Modern Pig Breeds Based on Fatty Acid Composition

**Figure 1:**
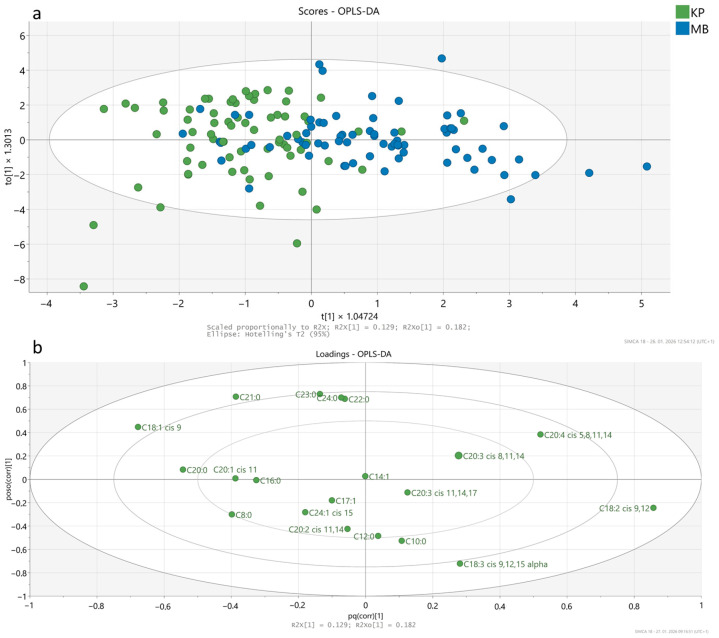
OPLS-DA model differentiating pig breeds (Krškopolje vs. modern) based on significantly different fatty acids (*n* = 20): (**a**) score plot illustrating sample clustering by breed—Krškopolje (KP) and modern (MB)—with a 95% confidence interval ellipse; (**b**) loading plot highlighting the key fatty acids that contributed most to sample clustering.

The OPLS-DA model built using significantly different fatty acids (*n* = 20) demonstrated a partial discrimination between meat samples from the Krškopolje (KP) and modern pig breeds (MB) (Figure 1a). The model showed an R2 of 0.380, a Q2 of 0.282, and an accuracy of 82.8%. Permutation testing (*n* = 200) confirmed that the observed separation is better than expected by chance (R2 intercept = 0.065, Q2 intercept = −0.101), indicating that the model captures true breed-related trends. However, the weak Q2 value reflects limited predictive ability, and separation should therefore be interpreted as partial.

Sample clustering along the predictive component t[1] indicated a tendency toward separation, with Krškopolje samples mainly distributed on the negative side and modern breed samples on the positive side of the score plot. Most samples were located within the 95% Hotelling’s T^2^ confidence ellipse, although a few outliers were observed. However, a degree of overlap between the two groups was observed, suggesting that fatty acid composition is influenced not only by genetic background but also by production system, feeding regime, and intra-breed variability, especially within modern breeds that encompass multiple genetic lines [23].

The loading plot (Figure 1b) identified fatty acids that contributed most to breed discrimination. The most influential fatty acids included C18:2 cis-9,12, C18:1 cis-9, C20:4 cis-5,8,11,14, C20:0, C21:0, C18:3 cis-9,12,15 (α-linolenic acid), and C20:1 cis-11. Overall, although only limited separation between breeds was achieved, OPLS-DA analysis revealed breed-specific trends in fatty acid composition, highlighting the influence of multiple factors on meat quality.

#### 3.1.2. Fatty Acid Composition of Krškopolje vs. Modern Breed Pig Meat in Relation to Rearing System (Organic vs. Conventional)

**Figure 2:**
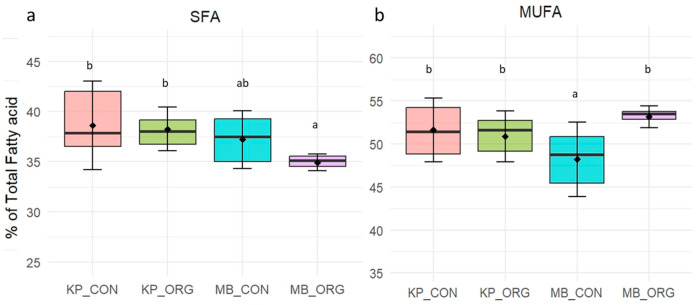
(**a**) Saturated fatty acid (SFA) and (**b**) monounsaturated fatty acid (MUFA) composition (% of total fatty acids) of meat from Krškopolje (KP) and modern pig breeds (MB) reared under organic (ORG) and conventional (CON) production systems. Different letters indicate significant differences (*p* < 0.05) within each fatty acid group.

**Figure 3:**
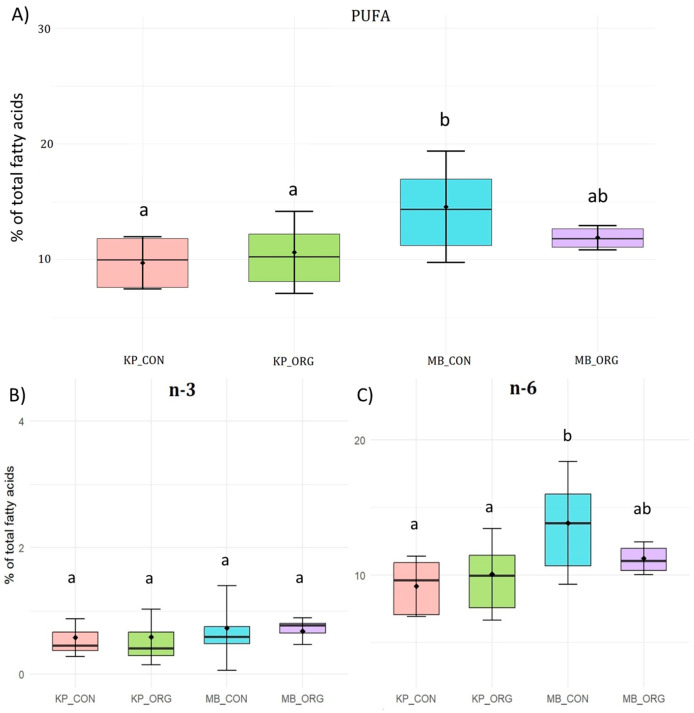
(**A**) Polyunsaturated fatty acids (PUFA), (**B**) omega-3 fatty acids (n-3), and (**C**) omega-6 fatty acids (n-6) composition (% of total fatty acids) in meat from Krškopolje (KP) and modern pig breeds (MB) reared under organic (ORG) and conventional (CON) production systems. Different letters indicate significant differences (*p* < 0.05) within each fatty acid group.

The fatty acid composition of pork meat was also influenced by the rearing system (Figure 2 and Figure 3). Across breeds, organic rearing was associated with lower SFA content than conventional production, especially in modern breeds (Figure 2a). The highest SFA levels were observed in Krškopolje pigs reared under conventional conditions (KP_CON), whereas the lowest values occurred in organically reared modern pigs (MB_ORG). The present study showed that organic meat from the modern breed had 6.4% lower SFA than conventionally produced modern breed meat, whereas no significant differences were observed within the Krškopolje breed. The reduction in SFA under organic rearing is likely related to differences in diet composition, notably lower energy density, as well as increased livestock physical activity [9]. According to Regulation 2018/848, organic livestock production, which mandates outdoor access and greater space allowance for animals, promotes greater physical activity than conventional indoor systems [9]. These requirements can be associated with altered lipid metabolism, reduced SFA deposition, and a shift toward a more favourable fatty acid profile in pork meat.

Organic rearing was also associated with higher MUFA proportions (by 9.9%) in modern breed pigs (MB_ORG) compared to conventionally reared modern pigs (Figure 2b). In contrast, the Krškopolje breed did not exhibit significant differences in SFA, MUFA, or PUFA content between organic and conventional systems, suggesting that the pigs of this breed remain predominantly reared on small-scale, non-intensive farms, very often on organic and agro-touristic farms [26]. This finding contrasts with Zybert’s [13] results, which reported greater differences between organic and conventional pork in traditional pig breeds.

PUFAs were affected by the rearing system only in the modern breed, with conventionally reared modern pigs exhibiting 20.4% higher total PUFA levels than organically reared pigs, primarily due to increased n-6 PUFA content (Figure 3A–C). This finding is in agreement with the results of Karwowska & Dolatowski [28] and Wójciak et al. [29], who reported a lower proportion of PUFAs in organic pork than in conventional pork. Moreover, Högberg et al. [30] reported that conventional feed contained higher PUFA levels than organic feed. This pattern is consistent with the composition of conventional diets, which are typically based on cereals and soybean meal and are rich in linoleic acid, thereby promoting PUFA accumulation in muscle lipids [18]. Notably, PUFAs are incorporated into the pig muscle directly from the diet, whereas MUFAs may be deposited either from the feed or by desaturation of SFAs [2].

Contrary to the pattern observed in the present study, other studies have reported higher PUFA content in organic diets than in conventional diets, especially when grain legumes or oilseed press cakes are included, as these cost-effective and widely available feed ingredients provide high-quality protein, essential amino acids, and additional dietary energy from residual fat [31,32]. However, pig breeds may respond differently to organic nutrition and environmental conditions due to breed-specific differences in lipid metabolism, fat deposition, and nutrient utilization, thereby altering fatty acid levels [32]. Furthermore, organic diets, regulated under EU legislation (Regulation 2018/848), restrict the use of synthetic amino acids and products from genetically modified organisms, while emphasizing organically produced feedstuffs, which may limit excessive n-6 PUFA deposition [9]. All of these factors may influence the composition and quality of fresh pork [5].

Overall, the findings suggest that breed influences the response to rearing system, with modern pigs being more sensitive to production conditions than Krškopolje pigs. It should be noted that fatty acid analysis of the organically reared modern breed samples was based on only four samples, limiting statistical power and potentially affecting the interpretation of breed- and production system-related differences.

### 3.2. Volatile Organic Compound (VOC) Profiles in Meat Samples from Krškopolje vs. Modern Breed

VOC analysis was performed on a subset of samples from only one production system per breed, and observed differences should not be interpreted as purely breed-specific. Relative mean percentages of the main VOCs are presented in Table 2. Overall, both breeds exhibited similar profiles, with some notable differences in specific compounds.

In both breeds, the predominant VOCs were 2-pentanol, 4-methyl, n-hexadecanoic acid (palmitic acid), and hexadecanal. Krškopolje breed meat exhibited a significantly higher relative percentage of hexadecanal, a long-chain aldehyde, derived from the degradation of unsaturated fatty acids (USFAs) and contributing to the grilled flavour of meat. The occurrence of hexadecanal in both breeds suggests similar oxidative processes affecting SFAs, likely influenced by comparable postmortem metabolism and storage conditions [33].

Several aldehydes, including hexanal, nonanal, octanal, and heptanal, did not differ significantly between breeds (*p* > 0.05). Saturated aldehydes had fruity, ethereal, nutty, and fresh flavour characteristics [34]. Furthermore, hexanal and heptanal are oxidation products of linoleic acid and arachidonic acid, respectively. At the same time, octanal and nonanal were produced by the oxidation of oleic acid, which gives pork meat its green grass aroma [35].

Several unsaturated aldehydes showed significant differences between breeds. 2-Undecenal (*E*) and 2-decenal (*E*) were higher in Krškopolje meat, whereas 2,4-decadienal (*E*,*E*) was significantly higher in modern breeds. These compounds are known contributors to meat flavour, often responsible for fatty, green, and roasted notes, indicating breed-specific differences in aroma precursors [36].

In the present study, benzaldehyde, characterized by bitter almond, fruity, and vanilla-like aroma notes, was more abundant in Krškopolje pork than in modern breed pork. However, the difference between breeds was not statistically significant. Benzaldehyde can be derived from the degradation product of phenylalanine through the Strecker degradation reaction [36].

Long-chain fatty acids, such as n-hexadecanoic acid and octadecanoic acid, were similar between breeds (*p* > 0.05). Hexadecanoic acid is commonly detected in pork meat; its presence is important as a precursor for aldehydes and alcohols generated during thermal processing and oxidative degradation [33]. Significant differences were observed for benzoic acid, which was higher in Krškopolje meat, and n-caproic acid vinyl ester, which was higher in modern breeds. These variations may reflect subtle differences in lipid metabolism or breed-specific characteristics [37].

Several phenolic compounds were detected exclusively, including phenol, 4-methyl, phenol, 3-methyl and phenol, 2-methoxy, or were present in significantly higher amounts, such as phenol, 4-(1-methylpropyl) (*p* = 0.023) in modern breeds. In addition, phenylethyl alcohol was detected only in modern breed meat. Ahamed et al. [33] also reported that the presence of an aromatic phenolic compound, phenol, 4-methyl, which is a metabolite derived from the breakdown of the amino acids tyrosine and phenylalanine, was found in pork.

3-Octanone; 2-pentadecanone; 2-heptanone, 6-methyl-; 2-nonanone, and 5,9-undecadien-2-one, 6,10-dimethyl- are ketones formed as a result of USFA oxidation and amino acid metabolism [34]. Li et al. [37] reported that 2-heptanone plays an important role in altering the volatile flavour of meat and meat products and can serve as a marker of product deterioration. Moreover, some ketones exhibit floral and fruity flavours, while diketones usually exhibit meaty and buttery flavours, depending on chain length and degree of saturation [34]. Acetoin, detected only in modern breed meat, is a short-chain hydroxyketone produced via microbial fermentation or pyruvate metabolism and is associated with buttery or creamy aromas [34].

Several VOCs were detected solely in meat from modern pig breeds, suggesting breed-specific metabolic profiles. Dodecanal, decanal, and 9-hexadecenal are long-chain aldehydes formed mainly via lipid oxidation of USFAs. These compounds typically contribute fatty, citrus, or green aroma notes, enhancing the overall flavour complexity of meat [36]. 1-Butanol, 3-methyl-; 1-nonanol, and 2-heptanol, 6-methyl- are primarily formed through amino acid metabolism or microbial activity during postmortem ageing.

Furthermore, phenylethyl alcohol was also detected only in modern breed meat and is an aromatic alcohol derived from phenylalanine catabolism, associated with floral and honey-like odours [36]. The presence of these VOCs exclusively in modern pig breeds suggests breed-specific differences in lipid composition, amino acid metabolism, or microbial interactions during postmortem ageing.

**Figure 4:**
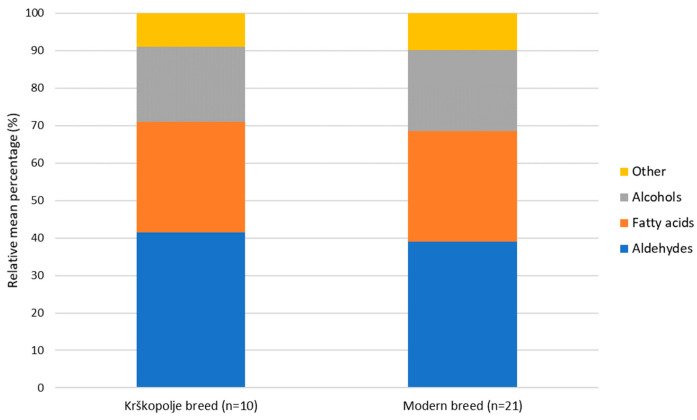
Relative mean percentages of the main groups of volatile organic compounds identified in meat from Krškopolje and modern pig breeds.

Aldehydes were the dominant VOC class in both breeds (Figure 4), with the Krškopolje breed having slightly higher levels, suggesting a greater extent of lipid oxidation or differences in lipid composition compared with modern breeds. Aldehydes are primarily formed through the oxidative degradation of fatty acids, particularly during postmortem metabolism, storage, and thermal processing, and they are key contributors to the characteristic fatty and green aroma of pork [38]. They also act as important intermediates in the Maillard reaction or lipid oxidation reaction and can participate in interactions between amino acids and carbonyl groups [36].

Fatty acids accounted for the second-largest group, suggesting comparable oxidative pathways in both breeds. Alcohols likely arising from amino acid metabolism and lipid oxidation contribute subtle fruity, malty, and floral notes [34]. The remaining minor compounds, including ketones, phenols, and other aromatic compounds, accounted for about 10% of the total profile.

### 3.3. Discrimination Between Krškopolje vs. Modern Pig Breeds Based on Volatile Organic Compound (VOC) Profiles

**Figure 5:**
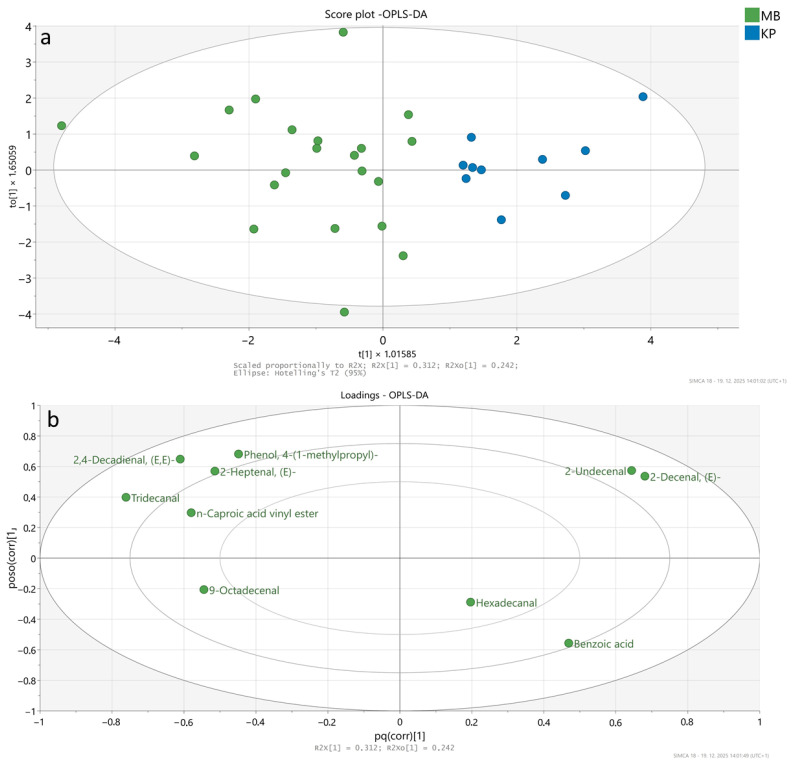
OPLS-DA model differentiating pig breeds (Krškopolje vs. modern) based on significantly different volatile organic compounds (*n* = 10): (**a**) score plot illustrating sample clustering by breed—Krškopolje (KP) and modern (MB)—with a 95% confidence interval ellipse; (**b**) loading plot highlighting the key VOCs that contributed most to sample clustering.

An OPLS-DA model (Figure 5) was created using 10 significant different VOCs (variables) and 31 samples to differentiate between Krškopolje and modern-breed meat. The model showed an R2 of 0.312, a Q2 of 0.456, and 100% apparent accuracy based on the training set. However, permutation testing (*n* = 200) yielded low R2 (0.0823) and negative Q2 (−0.424), indicating limited predictive reliability and suggesting that the apparent separation may be overoptimistic due to the small sample size.

The score plot (Figure 5a) shows a clear separation between Krškopolje and modern breed samples along the primary component (t[1]), indicating distinct VOC profiles between the two breeds. Krškopolje breed samples cluster predominantly on the right side of the plot, whereas modern breed samples cluster on the left, with minimal overlap. This separation implies that the VOC composition in meat differs between traditional Krškopolje pigs and modern breeds, likely reflecting differences in genetics, diet, or metabolism. The loading plot (Figure 5b) identifies the key VOCs that contribute most to the observed separation between breeds. VOCs positioned farther from the origin have the strongest influence on sample differentiation.

For modern breed samples, compounds such as tridecanal, 2,4-decadienal (*E*,*E*), n-caproic acid vinyl ester, phenol derivatives and 2-heptenal (*E)* were positively associated, suggesting higher relative abundance in modern breed pigs. 2,4-Decadienal (*E*,*E*) is formed through the oxidation of PUFAs, particularly linoleic acid (C18:2, n-6), and is commonly generated during meat cooking or storage as a result of lipid peroxidation. In contrast, VOCs such as 2-undecenal, 2-decenal (*E*), benzoic acid, and hexadecanal are positioned closer to Krškopolje breed samples, indicating that these compounds are more characteristic of the Krškopolje breed.

The OPLS-DA model discriminates between Krškopolje and modern pig breeds based on statistically significant VOCs, highlighting specific compounds that may underlie breed-specific aroma and flavour traits. The identified VOCs may represent promising candidate markers for breed differentiation or for exploring breed-related effects on meat quality and flavour profile; however, their application in authentication requires substantial further validation, including larger and independent cohorts, clearer separation of breed and production system effects, and evaluation of robustness across years and feeding conditions. These findings nevertheless suggest that autochthonous breeds, such as the Krškopolje breed, may retain distinct biochemical characteristics compared with intensively selected modern breeds.

A limitation of this study is the unequal sample sizes between breeds and production systems, particularly for VOC analysis, where fewer Krškopolje samples were available and only one production system per breed was represented. While this may reduce statistical power and limit the ability to separate breed and system effects, the selected samples were representative, and the observed fatty acid and VOC patterns are consistent with previously reported breed-specific traits, supporting the robustness of our results. Future studies with balanced sample sizes across breeds and production systems would further strengthen these findings.

## 4. Conclusions

To date, limited research has examined the fatty acid composition and aroma profiles of autochthonous pig breeds in comparison with modern breeds, as well as the influence of different production systems. The present results demonstrate that meat from the Krškopolje pig is characterized by higher SFA and MUFA contents, reflecting its traditional genotype and distinct lipid metabolism. In contrast, meat from modern pig breeds exhibited higher levels of PUFAs, particularly n-6 fatty acids, largely as a consequence of genetic selection and dietary practices. Furthermore, the results indicate that breed influences the response to rearing system, with the fatty acid composition of modern breed meat showing a stronger response to production conditions than that of the Krškopolje breed.

The presence of several VOCs in modern pig meat but not in the Krškopolje breed suggests breed-specific differences in lipid composition, amino acid metabolism, or microbial activity during postmortem ageing. Aldehydes were the dominant VOC class in both breeds; however, slightly higher levels were observed in the Krškopolje breed. Finally, the OPLS-DA model successfully discriminated between Krškopolje and modern pig breeds based on statistically significant VOCs, identifying key compounds that likely contribute to breed-specific aroma and flavour characteristics. Overall, these findings highlight the distinctive meat quality of the Krškopolje breed and demonstrate how breed and production system influence fatty acid and aroma profiles, providing a scientific basis for preserving autochthonous pig breeds.

## Figures and Tables

**Table 1:** Percentage of fatty acids of meat from Krškopolje and the modern pig breed, independent of rearing system.

Fatty Acid (% of Total Fatty Acid)	Krškopolje Breed	Modern Breed	Kruskal–Wallis (*p*-Value *)
SFA			
C8:0	0.01 ± 0.01	0.01 ± 0.01	0.051
C10:0	0.13 ± 0.09	0.14 ± 0.06	0.001
C12:0	0.12 ± 0.09	0.12 ± 0.07	0.012
C14:0	1.64 ± 0.64	1.61 ± 0.32	0.359
C15:0	0.06 ± 0.06	0.05 ± 0.07	0.515
C16:0	24.79 ± 1.52	24.23 ± 1.67	0.017
C17:0	0.28 ± 0.1	0.27 ± 0.07	0.582
C18:0	11.22 ± 2.52	10.56 ± 1.73	0.069
C20:0	0.16 ± 0.06	0.12 ± 0.05	<0.0001
C21:0	0.03 ± 0.03	0.00 ± 0.01	<0.0001
C22:0	0.04 ± 0.05	0.02 ± 0.05	0.001
C23:0	0.01 ± 0.01	0.01 ± 0.02	0.002
C24:0	0.05 ± 0.07	0.03 ± 0.09	0.002
∑SFA	38.54 ± 3.02	37.17 ± 2.43	0.011
MUFA			
C14:1	0.05 ± 0.12	0.03 ± 0.08	0.001
C16:1	3.71 ± 1.13	3.32 ± 0.72	0.060
C17:1	0.46 ± 1.31	0.34 ± 0.13	0.035
C18:1 trans 9	0.24 ± 0.12	0.24 ± 0.08	0.307
C18:1 cis 9	43.33 ± 2.97	40.5 ± 3.69	<0.0001
C18:1 n-7	3.95 ± 0.82	4.04 ± 0.88	0.309
C20:1	0.69 ± 0.36	0.57 ± 0.22	0.001
C24:1	0.23 ± 1.07	0.06 ± 0.13	<0.0001
∑MUFA	52.66 ± 3.71	49.1 ± 3.87	<0.0001
PUFA			
n-3 PUFA			
C18:3 (alpha)	0.44 ± 0.33	0.48 ± 0.21	0.012
C20:3 cis 11.14.17	0.08 ± 0.06	0.12 ± 0.57	0.046
C20:5 (EPA)	0.04 ± 0.06	0.05 ± 0.06	0.477
C22:6 (DHA)	0.05 ± 0.13	0.05 ± 0.07	0.319
n-6 PUFA			
C20:2	0.34 ± 0.44	0.33 ± 0.11	0.010
C20:3 cis 8.11.14	0.17 ± 0.29	0.21 ± 0.12	<0.0001
C20:4	1.09 ± 1.15	1.57 ± 1	0.000
C18:2	7.58 ± 2.26	11.23 ± 3.7	<0.0001
C18:3 (gamma)	0.37 ± 0.51	0.16 ± 0.26	0.577
∑n-3 PUFA	0.61 ± 0.36	0.7 ± 0.61	0.017
∑n-6 PUFA	9.55 ± 2.64	13.5 ± 3.85	<0.0001
n-6:n-3	15.7	19.3	
∑PUFA	10.16 ± 2.66	14.2 ± 3.89	<0.0001
AI	0.52 ± 0.08	0.50 ± 0.06	0.044
TI	1.19 ± 0.17	1.12 ± 0.15	0.009

SFA: saturated fatty acids; MUFA: monounsaturated fatty acids; PUFA: polyunsaturated fatty acids; n-3: omega-3 fatty acids; n-6: omega-6 fatty acids; AI: atherogenic index; TI: thrombogenic index. * Significant differences were considered at *p* < 0.05.

**Table 2:** Relative mean percentages of the main volatile organic compounds (VOCs) identified in meat from Krškopolje and modern pig breeds, independent of rearing system.

VOC	Krškopolje Breed (*n* = 10)	Modern Breed (*n* = 21)	Kruskal–Wallis *p*-Value *
hexanal	3.12	3.80	0.422
n-hexadecanoic acid	11.88	12.59	0.422
nonanal	3.72	4.62	0.375
octadecanoic acid	3.39	3.37	1.000
octanal	1.97	1.92	0.866
1-octanol	1.16	1.30	0.509
1-octen-3-ol	1.22	2.28	0.059
2-octenal, (*E*)-	0.72	0.96	0.218
benzaldehyde	3.48	2.95	0.083
benzoic acid	0.38	0.29	**0.048**
heptadecanal	1.05	1.41	0.057
hexanoic acid	0.54	0.35	0.059
octanoic acid	0.41	0.51	0.163
oleic acid	7.39	6.63	0.176
pentadecanal-	2.52	2.08	0.176
tetradecanoic acid	1.07	1.08	0.866
2-undecenal	1.90	1.15	**0.009**
9,12-octadecadienoic acid (*Z*,*Z*)-	0.95	1.14	0.312
2,4-decadienal, (*E*,*E*)-	0.40	1.05	**0.002**
2-decenal, (*E*)-	2.01	1.32	**0.010**
1-heptanol	0.68	0.57	0.910
acetic acid	0.53	0.56	0.141
tetradecanal	0.88	1.22	0.253
13-octadecenal	0.42	0.64	0.190
9-octadecenal	1.29	1.96	**0.025**
heptanal	0.44	0.43	0.639
nonanoic acid	0.33	0.26	0.153
2-nonenal, (*E*)-	1.05	0.75	0.221
9-decenoic acid	0.24	0.20	0.116
n-decanoic acid	0.94	1.01	0.735
2-pentanol, 4-methyl-	16.94	17.48	0.612
furan, 2-pentyl-	0.26	0.67	0.073
hexadecanal	14.32	10.08	**0.031**
octadecanal	2.22	2.77	0.447
palmitoleic acid	1.43	1.36	0.217
n-caproic acid vinyl ester	0.33	0.83	**0.016**
1-hexanol	0.23	0.24	0.665
1-pentanol	0.26	0.34	0.248
2-octen-1-ol	0.25	0.48	0.068
2,4-nonadienal, (*E*,*E*)-	0.19	0.27	0.593
2-heptenal, (*E*)-	0.31	0.75	**0.005**
1,3-hexadiene, 3-ethyl-2-methyl-	0.21	0.33	0.110
phenol, 4-(1-methylpropyl)-	0.27	0.76	**0.023**
1-dodecanol	nd	0.32	–
benzaldehyde, 3-ethyl-	0.31	0.37	0.643
benzaldehyde, 4-pentyl-	0.24	0.59	0.247
butanoic acid	0.29	0.25	0.564
1-hexadecanol	0.20	0.36	0.192
acetoin	nd	1.36	–
phenol, 4-methyl-	nd	0.15	–
3-octanone	nd	0.13	–
9-hexadecenal	nd	0.27	–
benzene, 1,2,3-trimethyl-	nd	0.15	–
dodecanoic acid	0.71	0.61	0.380
tridecanal	0.24	0.54	**0.020**
acetaldehyde	0.19	0.11	0.064
pentadecanoic acid	0.19	0.19	0.770
dodecanal	nd	0.64	–
1-butanol, 3-methyl-	nd	1.71	–
decanal	nd	0.23	–
tetradecane	0.17	0.18	1.000
1-nonanol	nd	0.28	–
2-pentadecanone	nd	0.13	–
butanoic acid, 3-methyl-	nd	0.43	–
phenylethyl alcohol	nd	1.32	–
2-heptanol, 6-methyl-	nd	0.18	–
2-heptanone, 6-methyl-	nd	0.10	–
2-nonanone	nd	0.15	–
5,9-undecadien-2-one, 6,10-dimethyl-	nd	0.14	–
nonadecane	nd	0.25	–
phenol, 2-methoxy-	nd	0.18	–
phenol, 3-methyl-	nd	0.12	–

nd = not detected; indicates concentrations below the established detection limit (LOD); *p*-values were calculated only for compounds detected in both groups. * Significant differences were considered at *p* < 0.05. Bold values indicate statistically significant differences (*p* < 0.05).

## Data Availability

The original contributions presented in the study are included in the article/Supplementary Materials. Further inquiries can be directed to the corresponding author.

## Supplementary Materials

The following supporting information can be downloaded at: https://www.mdpi.com/article/10.3390/foods15050866/s1, Figure S1: Variable Importance in Projection (VIP) plot highlighting the most influential variables (VIP > 1, red dashed line) for discrimination of meat from Krškopolje and modern pig breeds.

## Abbreviations

The following abbreviations are used in this manuscript:

SFA	Saturated fatty acids
USFA	Unsaturated fatty acids
MUFA	Monounsaturated fatty acids
PUFA	Polyunsaturated fatty acids
n-3	Omega-3 fatty acids
n-6	Omega-6 fatty acids
VOC	Volatile organic compounds
OPLS-DA	Orthogonal Partial Least Squares Discriminant Analysis
AI	Atherogenic index
TI	Thrombogenic index
