# Supplementary material for: Fatty Acid Composition and Aromatic Profile of Krškopolje and Modern Pig Breeds Reared Under Organic and Conventional Systems
Source: Foods. 2026 Mar 4;15(5):866. doi: 10.3390/foods15050866 (PMC12985004; doi:10.3390/foods15050866)
Supplement: Supplementary file 1 [file foods-15-00866-s001.zip › foods-4171491-supplementary.pdf]

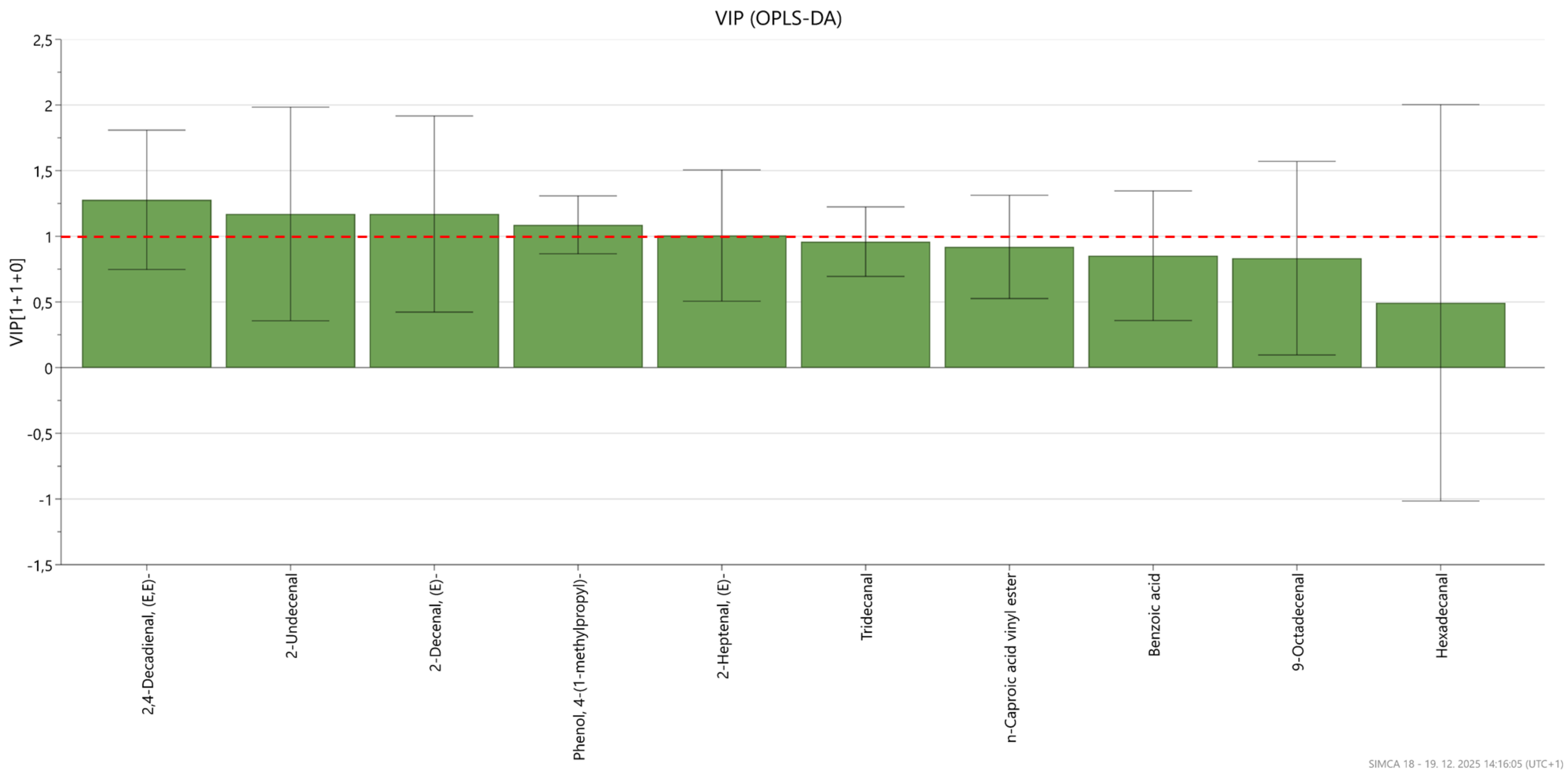

Figure S1. Variable Importance in Projection (VIP) plot highlighting the most influential variables (VIP > 1, red dashed line) for discrimination of meat from Krškopolje and modern pig breeds.
